# Untersuchungen zum Schädigungspotenzial durch den Konsum von E-Zigaretten: In-vitro-Exposition von humanen Nasenschleimhautzellen mit Propylenglykol im Air-Liquid-Interface

**DOI:** 10.1007/s00106-021-00998-2

**Published:** 2021-02-14

**Authors:** F. Wiest, A. Scherzad, P. Ickrath, N. Poier, S. Hackenberg, N. Kleinsasser

**Affiliations:** 1 Klinik und Poliklinik für Hals‑, Nasen- und Ohrenkrankheiten, plastische und ästhetische Operationen, Universitätsklinikum Würzburg, Josef-Schneider-Straße 11, 97080 Würzburg, Deutschland; 2 Klinik für Hals‑, Nasen- und Ohrenheilkunde, Kepler Universitätsklinikum Linz, Krankenhausstraße 9, 4021 Linz, Österreich

**Keywords:** Propylenglykol, Zytotoxizität, Genotoxizität, Entzündungsreaktion, Air-Liquid-Interface, Propylene glycol, Cytotoxicity, Genotoxicity, Inflammatory response, Air–liquid interface

## Abstract

**Hintergrund:**

Die E‑Zigarette erfreut sich in den letzten Jahren zunehmender Beliebtheit. Die Frage nach der Toxizität ist jedoch noch nicht eindeutig geklärt, und es herrscht global Unsicherheit im Umgang mit der E‑Zigarette.

**Ziel:**

Ziel der vorliegenden Arbeit war es, Propylenglykol, ein Hauptbestandteil der Liquide, in Bezug auf mögliche akute Entzündungsreaktionen, zyto- und genotoxische Auswirkungen auf humane Nasenschleimhautzellen zu untersuchen.

**Material und Methoden:**

Die Nasenschleimhautzellen wurden von zehn Probanden im Air-Liquid-Interface kultiviert und anschließend mit unterschiedlichen Konzentrationen des Propylenglykols bedampft. Die Analyse erfolgte mittels Trypanblau-Test, Comet-Assay, Mikrokerntest und IL-6- und IL-8-Sandwich-ELISA.

**Ergebnis:**

Der Trypanblau-Test zeigte keine Reduktion der Vitalität. Im Sandwich-ELISA konnte kein Anstieg der IL-6- und IL-8-Konzentrationen nachgewiesen werden. Im Comet-Assay zeigte das Olive Tail Moment eine Schädigung im Vergleich zur Negativkontrolle in allen untersuchten Konzentrationen. Zudem zeigte sich eine dosisabhängige Schädigung. Im Mikrokerntest konnte ein Unterschied zwischen dem Reinstoff und der Negativkontrolle gefunden werden.

**Schlussfolgerung:**

Es zeigten sich möglicherweise reparable DNS-Schädigungen im Comet-Assay. Im Mikrokerntest konnten diese nur in der Reinstoffkonzentration bestätigt werden. Es sollte ein restriktiver Umgang mit der E‑Zigarette erfolgen, bis insbesondere Langzeitstudien vorliegen. Zudem ist eine eindeutige Deklaration der Inhaltsstoffe der Liquide durch die Hersteller zu fordern, um weitergehende Schädigungspotenziale untersuchen zu können.

## Abstract (en)

**Background:**

The e‑cigarette has become increasingly popular in recent years. However, the question of toxicity is not yet clear and there is global uncertainty regarding the use of e‑cigarettes. This is intensified by the fact that there is a lack of declaration of the liquid ingredients.

**Objective:**

The present paper investigates propylene glycol, a major component of the liquids, for possible acute inflammatory reactions as well as cytotoxic and genotoxic effects on human nasal mucosa cells.

**Materials and methods:**

The nasal mucosa cells from 10 volunteers were cultivated at the air–liquid interface and then exposed to different concentrations of propylene glycol. The analysis was carried out using the trypan blue test, comet assay, micronucleus test, and interleukin (IL)-6 and IL‑8 sandwich ELISA.

**Results:**

The trypan blue test showed no reduction in vitality. No increase in IL‑6 and IL‑8 concentrations were detected in the sandwich ELISA. In the comet assay, the Olive tail moment showed a dose-dependent increase in DNA fragmentation compared to the negative control at all examined concentrations. A difference between the pure substance and the negative control was shown in the micronucleus test.

**Conclusion:**

Possibly repairable dose-dependent DNA fragmentation and profound DNA alterations at high concentrations of propylene glycol warrant enhanced genotoxicological studies. These should include long-term exposure studies and assessment of further ingredients of the liquids. Consequently, the manufacturers need to be forced to declare the latter.

Im Jahr 1965 wurde die E‑Zigarette das erste Mal erwähnt und in den USA von Herbert A. Gilbert aus Beaver Falls in Pennsylvania zum Patent angemeldet [15]. In den folgenden Jahrzenten geriet sie jedoch wieder in Vergessenheit, bis der Pharmazeut Hon Lik 2003 die E‑Zigarette in Kooperation mit der Fa. Ruyan auf dem chinesischen Markt etablierte [14]. Aktuell erfreut sich die E‑Zigarette nun zunehmender Beliebtheit: So berichtet die World Health Organisation (WHO), dass sich der weltweite Jahresumsatz von E‑Zigaretten und deren Zubehör 2014 auf ca. 3 Mrd. US-Dollar belief. Zudem hat sich die Anzahl der E‑Zigaretten-Manufakturen in wenigen Jahren fast verdoppelt [35]. So könnte im Jahr 2023 der Umsatz auf ca. 27 Mrd. US-Dollar steigen [29]. Im März 2017 erschien im *Deutschen Ärzteblatt* eine Studie, nach der von 4002 befragten Personen jede achte Person angab, schon einmal eine E‑Zigarette konsumiert zu haben [7].

Die Haptik der E‑Zigarette besteht grundsätzlich aus folgenden drei Basis-Elementen: einem Akku, einem Liquiddepot mit integriertem Heizelement und einem Mundstück. In das Liquiddepot wird das zu konsumierende Liquid gefüllt und dient als Reservoir. Dabei kann der Konsument aus einer nahezu unbegrenzten Auswahl an Liquiden mit verschiedenen Geschmacksrichtungen und jeweiligen Konzentrationen des Nikotingehalts wählen. In Untersuchungen der Liquide auf ihre Bestandteile konnte festgestellt werden, dass Propylenglykol mit ca. 66 %, gefolgt von Glycerin mit ca. 24–40 %, die beiden Hauptbestandteile sind. In ihrem Volumen vernachlässigbar sind kleine Mengen an Wasser und Geschmacksstoffen [27]. Der Stoff Glycerin wurde bereits in unsrer eigenen Arbeitsgruppe hinsichtlich seiner Toxizität als Feuchthaltemittel von Shisha-Tabak untersucht. Dabei konnten geno- und zytotoxische Effekte in In-vitro-Studien beobachtet werden [31].

Die zentrale Frage nach der Toxizität der Liquide lässt sich mit der derzeitigen Studienlage noch nicht beantworten: Verschiedene humanmedizinische Untersuchungen zeigten eine Reduktion des Tiffeneau-Index, Augenreizungen und Dyspnoe [36]. Zusätzlich wurden noch Formaldehyd, Acetaldehyd, Acrolein und Nitrosamine mittels chromatographischen und spektroskopischen Methoden im Dampf nachgewiesen [16]. Ebenso konnten Nanopartikel von Feststoffen wie Eisen, Silber, Silikat, Nickel, Chrom und Zinn festgestellt werden [37]. Manche Forschergruppen befürworten die Verwendung der E‑Zigarette als probates Mittel zur Raucherentwöhnung, da die E‑Zigaretten zu einer geringeren Reduktion des Tiffeneau-Index führen als konventionelle Zigaretten [4].

Die hier vorgestellten Untersuchungen zielen in einem lebensnahen Modell mit einer Bedampfung humaner Schleimhaut auf den Hauptbestandteil der Liquide, das Propylenglykol. Es sollen folgende Fragen beantwortet und eventuell Handlungsempfehlungen zum Umgang mit der E‑Zigarette erarbeitet werden:

- Inwieweit ist Propylenglykol zellschädigend?
- Schädigt Propylenglykol die DNS?
- Erzeugt Propylenglykol einen Anstieg der Interleukin-6(IL-6)- und Interleukin-8(IL-8)-Konzentration und somit eine Entzündungsreaktion?
- Muss Propylenglykol als Gefahrenstoff gekennzeichnet werden und eventuell für die Nutzung in E‑Zigaretten eingeschränkt werden?

## Material und Methoden

In dieser In-vitro-Studie wurden Nasenschleimhautzellen von zehn Patienten der Klinik und Poliklinik für Hals‑, Nasen‑, Ohrenkrankheiten, plastische und ästhetische Operationen des Universitätsklinikums Würzburg verwendet. Die Proben fielen im Rahmen von medizinisch angeratenen Operationen zur Verbesserung der Nasenluftpassage an. Die Patienten wurden entsprechend den Empfehlungen der Ethikkommission der Medizinischen Fakultät (Az. 16/06) über die Studie aufgeklärt und erteilten ihre schriftliche Einwilligung.

### Probengewinnung und Kultivierung

Die gewonnen Schleimhautproben wurden zunächst von Blutresten, Knochen- und Knorpelresten befreit. Anschließend wurde die Proben mit einem Enzymmix von 100 µl und 9,9 ml Nährmedium für 24 h lichtgeschützt bei 4 °C auf einem Schüttler gemischt. Am nächsten Tag wurden 13 Wells mit jeweils 300 μl einer gleichteiligen Mischung aus PBS (Fa. Roche Diagnostics, Rotkreuz, Schweiz) und Kollagen A (Fa. Biochrom, Berlin) für 30 min inkubiert und wieder abgesaugt. Die Patientenprobe wurde nun in 2 ml fetalem Kälberserum (FCS; Fa. Biochrom, Heidelberg) aufgenommen, gefiltert und für 5 min bei 4 °C bei 500 g zentrifugiert. Nach Absaugen des Überstands wurde die Suspension mit 6,5 ml (500 μl pro Well bei 13 Wells) Airway Epithelial Cell Growth Medium (AEGM, Fa. PromoCell GmbH) mit Supplement und 1 % Penicillin/Streptomycin resuspendiert. In jedes Well wurden 500 μl der Zellsuspension pipettiert. Zuletzt wurde 1 ml des AEGM mit Zusätzen in die basalen und 500 μl in die apikalen Kompartimente gegeben. Bestand nach 7–9 Tagen der Kultivierung unter lichtmikroskopischer Kontrolle eine Konfluenz von mehr als 70 %, wurde nur noch das basale Kompartiment versorgt. Nach weiteren 3 Tagen der Kultivierung konnte die Exposition erfolgen.

### Exposition

Das zentrale Element der Dampfexposition in einem Air-Liquid-Interface (ALI) bildet die Expositionskammer (Fa. VitroCell Systems GmbH, Waldkirch). Diese wird an ein Wasserbad mit Umwälzpumpe (Fa. Thermo Scientific) angeschlossen und auf 37 °C erwärmt. Über den Abluftzugang der Expositionskammer wurde eine Pumpe vom Typ Trivac (Fa. Leybold, Köln) angeschlossen. Diese wird über einen zwischengeschalteten Massendurchflussregler (MFC-Regler, Fa. Bronkhorst High-Tech BV, AK Ruurlo (NL)) auf 20 ml/min eingestellt. Bei einer Sogstärke von 20 ml/min konnte der natürliche Widerstand des Mundstücks der E‑Zigarette überwunden werden, und es muss nicht mit Zellschädigung durch Austrocknung seitens des erhöhten Luftstroms gerechnet werden [1]. Die Zuluft der Expositionskammer wurde über ein Verteilerstück mit dem Verdampfer und Liquiddepot der E‑Zigarette verbunden. Daran wurde eine Zeitschaltuhr und ein Akku angebracht, um den speziellen Dampfexpositionen gerecht zu werden: Die Aktivierung der Heizspirale und somit die Verdampfung erfolgte 4 s lang, in einem Intervall von 30 s, über den gesamten Zeitraum von einer Stunde pro Konzentration. Dieser Zeitablauf wurde bereits mehrfach in In-vitro*-*Modellen verwendet und für diese Arbeit übernommen [9, 10]. Für jede Konzentration wurde eine eigene E‑Zigarette verwendet, um Kontaminationen zu vermeiden. Zudem wurde bei jeder Exposition ein neues Heizelement verwendet.

Die VitroCell-Expositionskammer wurde zunächst mit Ethanol und PBS vorgereinigt, danach die jeweiligen Konzentrationen des Propylenglykols (Fa. Sigma-Aldrich, Darmstadt) und die Positivkontrollen hergestellt. Für die einzelnen Propylenglykol-Konzentrationen wurde Aqua ad injectabilia (Fa. B. Braun Melsungen, Melsungen) verwendet, um den Filter der E‑Zigarette nicht durch Salze oder Proteine zu verunreinigen und somit ungewollte Verbrennungen und Schadstoffe zu erzeugen. Für jede untersuchte Konzentration wurde jeweils 1 Well für den Comet-Assay und den Mikrokerntest in die Expositionskammer eingesetzt. Zusätzlich wurde in jedes Kompartiment 1 ml AEGM mit 1 % Penicillin/Streptomycin ohne Supplement für die spätere IL-6- und IL-8-Bestimmung gegeben. Nach jeder Exposition wurden die Kammern mit PBS gereinigt.

### Einzelzell-Mikrogel-Elektrophorese-(Comet‑)Assay

Der Comet-Assay ist ein bereits etabliertes Verfahren zur Detektion von DNS-Schäden, welches nach der Verfahrensweise von Singh und Mitarbeitern in dieser Arbeit verwendet wurde [30]. Die exakte Durchführung und das Verfahrensprotokoll wurden bereits in einer vorausgegangenen Untersuchung unserer Arbeitsgruppe ausführlich dargestellt [3].

### Trypanblau-Test

Der Trypanblau-Test diente zur Vitalitätsbestimmung. Nun wurden 10 μl der exponierten Zellsuspensionen mit 10 μl Trypanblau vermischt. Daraus wurden 10 μl auf eine Neubauerzählkammer gegeben und lichtmikroskopisch bei 10-facher Vergrößerung ausgewertet: Für jede Konzentration wurde der Quotient aus den vitalen Zellen und der gesamten Zellzahl gebildet.

### Mikrokerntest

Mikrokerne sind eigenständig vom Nukleolus abgelöste Chromatin-Ansammlungen. Diese können unter anderem durch mutagene Substanzen oder ionisierende Strahlungen entstehen. Dabei können sich einzelne DNS-Stückchen bis hin zu ganzen Chromosomen abspalten und während der Mitose nicht mehr mittels des Spindelapparats in die Tochterzellen überführt werden [11]. Es wurden die bereits etablierten Kriterien nach Fenech als Definition für das Vorliegen eines Mikrokerns angewandt [12]. Die exakte Durchführung und das Verfahrensprotokoll wurden bereits in einer vorausgegangenen Untersuchung unserer Arbeitsgruppe ausführlich dargestellt [3].

### Sandwich-ELISA

Der „Enzyme-Linked Immunosorbent Assay“ (ELISA) ist ein sensitives Verfahren, um Antigene quantitativ zu messen. In dieser Arbeit sollte die IL-6- und -8-Konzentration als Hinweis auf eine akute Entzündungsreaktion nach Exposition untersucht werden. Dafür wurden nach Exposition die basalen Überstände des Mediums in Reaktionsgefäße (1,5 ml) überführt und bei −20 °C für eine spätere Analyse mit den Sandwich-ELISA-Kits der Fa. Diaclone (Besançon, Frankreich) eingefroren [6].

### Statistische Auswertung

Die statistische Auswertung erfolgte mit dem Software-Programm Statistica 13 (Quest, Köln). Dabei wurden der Friedman- und der Wilcoxon-Vorzeichen-Rang-Test (W-Test) angewandt. Das Signifikanzniveau wurde mit 0,05 definiert. Um einem Fehler der 1. Art beim multiplen Testen entgegenzuwirken, wurde die Bonferroni-Holm-Korrektur (BH-Korrektur) als Post-hoc-Test verwendet.

## Ergebnisse

**Figure 1:**
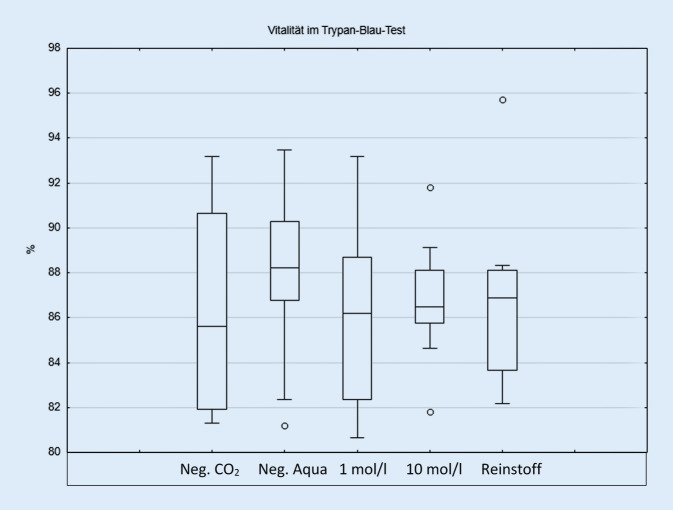

**Figure 2:**
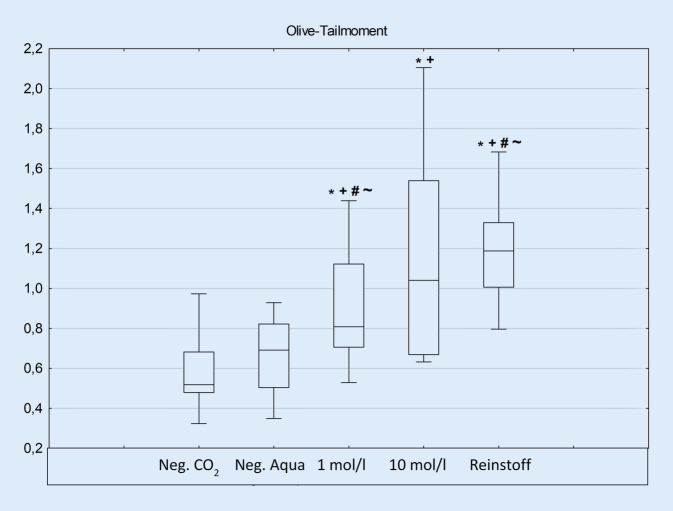

Bezüglich der Vitalität zeigte sich nach einstündiger Exposition keine Reduktion im Trypanblau-Test im Vergleich zur Negativkontrolle (Abb. 1). Ebenso konnte kein signifikanter Anstieg der IL‑6 oder IL-8-Konzentration gefunden werden (ohne Abb.). Im Comet-Assay war eine Schädigung im Vergleich zur Negativkontrolle in allen untersuchten Konzentrationen nachweisbar. Zudem zeigte sich eine dosisabhängige Schädigung der Konzentrationen 1 mol/l und Reinstoff (Abb. 2).

**Figure 3:**
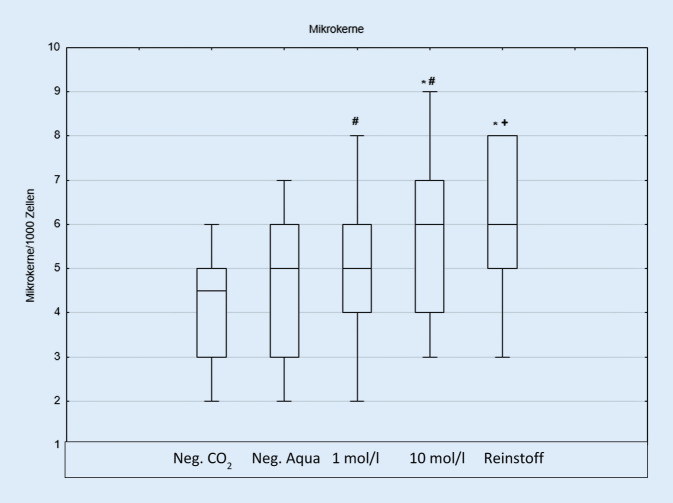

Im Mikrokerntest konnte ein signifikanter Unterschied zwischen dem Reinstoff und der Negativkontrolle gefunden werden. Eine dosisabhängige Schädigung konnte hier nicht gezeigt werden (Abb. 3).

## Diskussion

### Überlegungen zu den vorgelegten Untersuchungen

Das hier eingesetzte Testsystem basiert u. a. auf einer Modifikation der Vorarbeiten der eigenen Arbeitsgruppe [21]. Zwar konnte die Konzentration des Liquids im Liquiddepot individuell festgelegt, jedoch nicht gemessen werden: In der Expositionskammer selbst entzieht sich die Konzentration der Kontrolle. Damit eine Schädigung durch den Versuchsaufbau an sich ausgeschlossen werden konnte, wurde die Negativkontrolle Aqua mit der Negativkontrolle CO_2_ verglichen, die im Brutschrank im Rahmen der Exposition bei 5 % CO_2_ für eine Stunde lagerte. Die eingesetzten Testverfahren Comet-Assay, Mikrokerntest, Trypanblau-Test und Sandwich-ELISA sind bereits etablierte und valide Untersuchungsmethoden [12, 25, 29].

Die Ergebnisse dieser Arbeit zeigen, dass sich die Vitalität der exponierten Nasenschleimhautzellen gegenüber der Negativkontrolle nicht signifikant unterscheidet. Infolgedessen kann davon ausgegangen werden, dass Propylenglykol nicht zytotoxisch auf Nasenschleimhautzellen wirkt. Diese Ergebnisse gliedern sich sehr gut in die bereits bestehende Studienlage ein [25]. So konnte zudem durch unsere eigene Arbeitsgruppe gezeigt werden, dass auch andere Tabakstoffe wie Schnupftabak keinen Einfluss auf die Vitalität der exponierten Nasenschleimhaut haben [3].

Die Ergebnisse der vorliegenden Untersuchungen im Comet-Assay weisen jedoch auf eine genotoxische Schädigung der Nasenschleimhautzellen durch Propylenglykol hin. Ähnliches konnte in einer Studie mit humanen Lungenfibroblasten im ALI gezeigt werden. Die Expositionsparameter wurden wie in dieser Arbeit mit Zügen von 4 s und jeweils 30 s Pause gewählt. Jedoch war der untersuchte Zeitraum auf 5, 10, 15 und 20 min beschränkt und erbrachte außer in der 5‑min-Exposition signifikante Unterschiede im Vergleich zur Negativkontrolle [23]. Ähnliche Ergebnisse waren ebenfalls mit humanen Schleimhautzellen des Oropharynx zu beobachten, wobei das Ausmaß der Schädigung der Liquide mit Fruchtgeschmack wesentlich höher war als mit Tabakgeschmack [33]. Ebenfalls konnten Schäden in humanen Hautzellen (HaCat) und humanen Kopf-Hals-Plattenepithelkarzinomen mittels Comet-Assay nachgewiesen werden [38].

Ishidate und Mitarbeiter exponierten Fibroblasten von Hamstern mit Propylenglykol und analysierten die DNS-Schäden mittels Mikrokerntest. In Konzentrationen von 32 mg/ml (420,6 mmol/l) über 48 h Exposition stieg die Anzahl der Mikrokerne signifikant an. Hierbei ist zu bedenken, dass die osmotische Veränderung im Medium ursächlich für den resultierenden Schaden sein könnte [20]. In einer In-vivo*-*Folgestudie mit Mäusen konnte das Ergebnis nicht bestätigt werden. Dabei wurde das Propylenglykol intraperitoneal gespritzt und ausgespülte Knochenmarkzellen des Femurs untersucht [18].

Diese In-vitro-Ergebnisse können nicht eins zu eins auf In-vivo-Untersuchungen oder direkt auf den Menschen übertragen werden. Dafür müssen weitere Faktoren wie Immunsystem, Reparaturvorgänge und unspezifische Abwehrsystem wie Schleimproduktion der Nasenschleimhaut berücksichtigt werden. Doch konnten erste In-vivo-Studien mittels High-Speed-Kamera eine reversible Reduktion des Zilienschlags der Nasenschleimhautzellen und damit eine gestörte Integrität des Abwehrsystems nachweisen [32]. Zudem zeigten sich nach Langzeitexpositionen von 90 Tagen Metaplasien im Larynx von Hunden oder in der Lunge von Ratten [28, 34].

In der hier vorliegenden Arbeit waren nach einstündiger Exposition bis auf sechs Einzelergebnisse alle Interleukin-Konzentrationen unterhalb der Nachweisgrenze und konnten somit keinen Hinweis auf eine Entzündungsreaktion erbringen. Ähnliche Ergebnisse konnten mit humanen Lungenkarzinomzellen und Hamster-Ovarzellen bestätigt werden [26]. Dem gegenüber stehen verschiedene Studien, die eine signifikante Interleukin-Produktion nachweisen konnten. So wurden humane Lungenfibroblasten mittels ALI dem Dampf von E‑Zigaretten ausgesetzt. Dabei konnte 18 h nach Exposition im Vergleich zur Negativkontrolle ein signifikanter Anstieg des IL‑6 und -8 mittels des Dual-Antikörper-Kit nachgewiesen werden [23]. Cervelatti und Mitarbeiter konnten mit HaCat-Zellen und Lungenzellen ähnliche Ergebnisse erzeugen: Bei ihnen stieg unter anderem die IL-8-Konzentration in beiden Zellgruppen stark an, wohingegen die IL-6-Konzentration im Vergleich zur Negativkontrolle abnahm [4]. Eine Erklärung für den fehlenden Anstieg der Interleukine in der hier vorliegenden Arbeit könnte die direkte Bestimmung nach der Exposition und die fehlende Zeit für die Synthese der Proteine sein. In verschiedenen Studien konnte der Anstieg des IL‑6 im zeitlichen Verlauf betrachtet werden. So wurden nach verschiedenen Bauchoperationen an Menschen die IL-6-Konzentrationen immer wieder bestimmt und erst 3 h nach der Operation zeigten sich Veränderungen im Serum. Nach 24 h war der Zenit erreicht [26].

### Gesundheitspolitische Relevanz

Das Bundesinstitut für Risikobewertung (BFR) deklarierte im Jahr 2012 den Konsum der E‑Zigarette als ein nicht abschätzbares Risiko und riet vom Konsum ab [2]. Momentan ist der Stoff Propylenglykol durch die Europäische Chemikalienagentur (ECHA) nicht als Gefahrenstoff gekennzeichnet und ist im Anhang VI des „Classification, Labelling and Packaging“ (CLP) als nicht gesundheitsschädlicher Stoff gelistet [8]. Für Deutschland gibt es derzeit ebenfalls keinen Grenzwert für die maximale Arbeitsplatz-Konzentrations- (MAK) und die Biologische Arbeitsstoff-Toleranzwert-Werte-Liste (BAT) 2016 [5].

Auch in der deutschen Gesetzgebung fällt die E‑Zigarette bis zum heutigen Tag nicht unter den § 1 des Bundesnichtraucherschutzgesetzes (BNichtrSchG), da ihr Liquid verdampft und nicht wie bei der konventionellen Zigarette der Tabak verbrannt wird. Demzufolge darf sie an öffentlichen Orten, auch Kneipen und Bars, konsumiert werden. Es kann jedoch zu Einschränkungen der Nutzung durch das bestehende Hausrecht kommen.

Die Regierungsbehörde „Public Health England“, Exekutivagentur des „Department of Health and Social Care“ im Vereinigten Königreich, ist dem Konsum der E‑Zigarette gegenüber offener eingestellt. Diese Behörde deklariert in einer Stellungnahme, dass der Konsum der E‑Zigarette unbedenklich sei und durch die widersprüchlichen Forschungsergebnisse im Vergleich zur konventionellen Tabakzigarette als harmloser gelte [17, 25].

Laut der amerikanischen Gesundheitsbehörde „Centers for Disease Control and Prevention“ sind bis September 2019 in über 33 Bundesstaaten 450 mögliche Erkrankungen mit Atemnot und Brustkorbschmerzen und sechs verstorbene Personen durch den Konsum der E‑Zigarette registriert worden [13]. In vielen der Erkrankungsfälle wurde festgestellt, dass den Liquiden der E‑Zigarette zusätzlich Tetrahydrocannabinol, Wirkstoff des Cannabis, beigemischt wurde. Abschließende Untersuchungen stehen aktuell noch aus [24]. Bereits im September 2019 forderte der damalige Präsident der USA ein Teilverbot für den Verkauf bestimmter Liquide. Vor allem aromatisierte Liquide, die besonders junge Konsumenten ansprechen, wie Gurke, Mango und Minze, sollten komplett verboten werden, sodass auch Erwachsene diese nicht mehr erwerben können. Es sollten lediglich Liquide mit Tabakgeschmack erhalten bleiben [13, 24]. Der Präsident konnte seine strikten Forderungen nicht komplett verwirklichen: So wurde zwar das Mindestalter zum Kauf von E‑Zigaretten und deren Produkten von 18 auf 21 Jahre angehoben, jedoch gilt ein Verkaufsverbot nur für E‑Zigaretten, die bereits in ihrem Depot mit einem Fruchtliquid befüllt worden sind. Geschmacksrichtungen mit Tabak und Menthol unterliegen keinen Restriktionen, ebenso können E‑Zigaretten immer noch durch den Kunden eigenständig mit einem separat gekauften, beliebigen Liquid befüllt werden [22].

Die indische Regierung verabschiedete im Dezember 2019 ein gesetzliches Verbot, um junge Erwachsene vor den nicht absehbaren gesundheitlichen Folgen des Konsums der E‑Zigarette zu schützen. Dabei sind sowohl Produktion, Verkauf, Import und Transport unter Strafe gestellt worden. Das Strafmaß beläuft sich zwischen einmaligen Geldzahlungen bis hin zu mehrjährigen Gefängnisstrafen bei Wiederholungstätern [19].

Die globale Unsicherheit bezüglich der Frage, ob die E‑Zigarette ein geeignetes Ersatzprodukt für Tabakzigaretten ist, oder Jugendliche zum Rauchen und Dampfen verführt und damit gesundheitlich gefährdet, betrifft auch die deutsche und europäische gesundheitspolitische Debatte. Die hier vorgelegten Ergebnisse deuten auf ein relevantes Schädigungspotenzial durch den Konsum von E‑Zigaretten und indizieren Studien mit weiteren Endpunkten der Genotoxizität als möglichen Hinweis auf eine Induktion von Tumoren im oberen Aerodigestivtrakt.

## Fazit

Die vorgelegten Laboruntersuchungen weisen in der Zusammenschau mit der Literatur auf ein gesundheitsgefährdendes Potenzial durch den Konsum von E‑Zigaretten. Es fehlen bislang jedoch vielfältige Untersuchungen mit den variablen Inhaltsstoffen der käuflich erwerbbaren Liquide. Durch die derzeitige unsichere Studienlage ist der Gesundheitspolitik ein restriktiver Umgang mit E‑Zigaretten zu empfehlen und eine eindeutige Deklaration der Inhaltsstoffe der Liquide durch die Hersteller zu fordern.
